# Continuous Monitoring of Turning in Patients with Movement Disability

**DOI:** 10.3390/s140100356

**Published:** 2013-12-27

**Authors:** Mahmoud El-Gohary, Sean Pearson, James McNames, Martina Mancini, Fay Horak, Sabato Mellone, Lorenzo Chiari

**Affiliations:** 1 APDM, Inc., Portland, OR 97201, USA; E-Mails: seanp@apdm.com (S.P.); mcnames@apdm.com (J.M.); 2 Oregon Health & Science University, Portland, OR 97239, USA; E-Mails: mancinim@ohsu.edu (M.M.); horakf@ohus.edu (F.H.); 3 University of Bologna, Bologna 40126, Italy; E-Mails: sabato.mellone@unibo.it (S.M.); lorenzo.chiari@unibo.it (L.C.)

**Keywords:** Parkinson's disease, movement disability, continuous monitoring, turning, inertial sensors, gyroscopes, accelerometers

## Abstract

Difficulty with turning is a major contributor to mobility disability and falls in people with movement disorders, such as Parkinson's disease (PD). Turning often results in freezing and/or falling in patients with PD. However, asking a patient to execute a turn in the clinic often does not reveal their impairments. Continuous monitoring of turning with wearable sensors during spontaneous daily activities may help clinicians and patients determine who is at risk of falls and could benefit from preventative interventions. In this study, we show that continuous monitoring of natural turning with wearable sensors during daily activities inside and outside the home is feasible for people with PD and elderly people. We developed an algorithm to detect and characterize turns during gait, using wearable inertial sensors. First, we validate the turning algorithm in the laboratory against a Motion Analysis system and against a video analysis of 21 PD patients and 19 control (CT) subjects wearing an inertial sensor on the pelvis. Compared to Motion Analysis and video, the algorithm maintained a sensitivity of 0.90 and 0.76 and a specificity of 0.75 and 0.65, respectively. Second, we apply the turning algorithm to data collected in the home from 12 PD and 18 CT subjects. The algorithm successfully detects turn characteristics, and the results show that, compared to controls, PD subjects tend to take shorter turns with smaller turn angles and more steps. Furthermore, PD subjects show more variability in all turn metrics throughout the day and the week.

## 1. Introduction

The ability to alter our locomotor trajectory by turning safely is important for functional independence, but surprisingly much more difficult for the nervous system to control than straight-ahead walking. Difficulty turning during gait is a major contributor to mobility disability, falls and reduced quality of life in older people and people with movement disorders. Studies have found that measurements of turning discriminate elderly fallers from non-fallers [1]. Falls during turning are particularly dangerous, because they usually result in contact of the femur with the ground, which results in eight-times more hip fractures compared with falls during straight-ahead walking [2,3]. Although turning gradually becomes more difficult as we age due to increasing sensorimotor impairments, turning is particularly difficult for people with Parkinson's disease (PD). Studies have shown that PD increases the risk of falling by over 50%, and most falls occur while walking and turning [4,5]. Furthermore, freezing of gait (FoG), in which PD patients perceive their feet ‘glued to the floor’, most often occurs when attempting a turn [6–8].

Current clinical measures to assess mobility in PD and other movement disorders are coarse and insensitive. The most common objective method of tracking movement is based on optical Motion Analysis systems. However, these systems are costly, time consuming and can only be used in large laboratories. The need to overcome these limitations has led to an upsurge in research on the use of wearable inertial sensors for the study of human movement [9]. Sensors, including accelerometers and gyroscopes, have widely been used in wearable systems to quantify balance [10,11], characterize gait [12–15], evaluate fall risk [16] and detect FoG [17–21].

Gait research has focused primarily on straight-ahead walking [22,23], and the research on turning has been limited to laboratory or clinical investigations [24]. Recent studies have shown that objective measures of turning mobility are more sensitive than gait speed or clinical measures of mobility to detect impaired mobility in patients with mobility disorders [25,26]. More specifically, patients with PD or multiple sclerosis (MS) exhibit abnormal turning characteristics even though they have normal speed of straight-ahead walking [27]. Other studies have suggested that turning-related neural systems may be more vulnerable to impairments than a straight-ahead, linear gait. This is due to the fact that turning involves more interlimb coordination, more coupling between posture and gait and modifications of locomotor patterns requiring frontal lobe cognitive and executive function that plays a role in postural transitions [28,29].

The assessment of gait and balance in the clinic does not adequately reflect mobility function during daily life, and little is known about turning characteristics in the home or community settings. Neurologists currently rely upon patient interviews and unreliable diaries for medical decision-making regarding fluctuations in motor function. As PD advances, patients experience frequent fluctuations in their motor function. Movement disorder specialists spend much of their effort on reducing these fluctuations by adjusting medication schedules and dosages, changing medications and referring to deep brain stimulation surgery. A brief medical examination of PD patients misses these diurnal fluctuations.

Clinicians and patients would benefit from a system they can easily use to measure daily mobility and assess its fluctuations throughout the day, evaluate their risk of falling and measure the effects of treatment and exercise. However, no current system actually characterizes the quality of gait or turning or mobility fluctuations across days and weeks, because of the lack of sophisticated analysis and adequate technology. A few earlier studies to measure movement for long periods of time utilized activity monitors (Actigraphs) [30,31]. They monitor patient's activity cycles and provide a measure of step counts and the variability of walking time. Unfortunately, these activity monitors provide no information on the type or quality of movement. Rochester *et al.* used activity monitors (ActivePal) to quantify changes in ambulatory activity following deep brain stimulation in advanced PD over a seven-day period. They found a significant increase in the length and variability of walking bouts, but the total number of steps per day did not change [32]. Human motor activity has many measurable facets, besides step counts, that can identify fall risk. Novel measurement and analysis of turning characteristics will provide insights beyond the counts of gait bouts that are routinely used.

In this study, we use wearable inertial sensors to detect and analyze prescribed and spontaneous turns during gait in the laboratory and home. In addition to turning onset, the turn detection algorithm estimates other turn metrics, including duration, peak and mean velocity, number of steps to complete a turn and body jerk during a turn. We demonstrate the validity of our inertial algorithm in both the laboratory and home environment. In the laboratory, the sensitivity and specificity of the inertial algorithm is assessed using a Motion Analysis system and video data from a waist-mounted video camera aimed at the feet. We also evaluate the performance of the inertial algorithm during seven days of continuous data collected in subjects' homes. To the best of our knowledge, our study is the first to characterize spontaneous walking and turning in the home for an extended period of one week.

## 2. Methods

In order to develop and validate the accuracy and reliability of the turn detection algorithm, we collected two sets of data. The first set was collected in the Balance Disorders Laboratory at the Oregon Health and Science University (OHSU). A second set of continuous monitoring data was collected in subjects' homes throughout a period of seven days. The following section describes the subjects, data collection protocol, and the algorithm for detecting turns and corresponding metrics.

### 2.1. Measurement in the Laboratory

**Figure 1.**
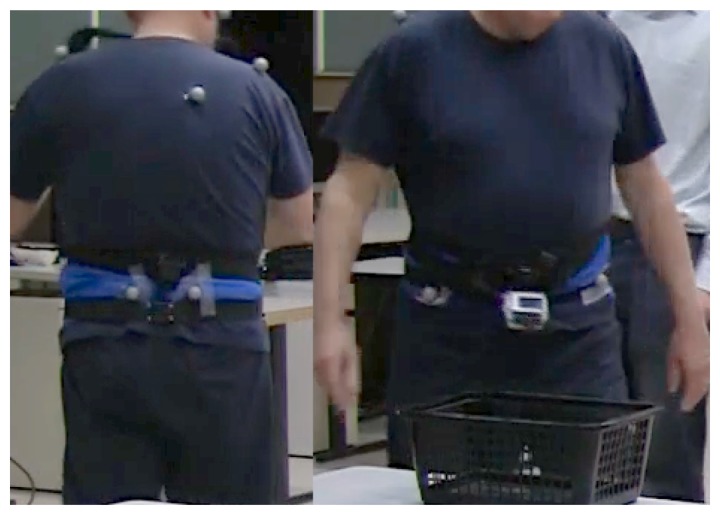
Inertial sensor, markers placement (back) and video camera attachment (front).

We examined 21 PD subjects (65 ± 6 years, Unified Parkinson's Disease Rating Scale (UPDRS) version III 24.5 ± 7.5) and 19 control subjects (67 ± 9 years) wearing an Opal inertial sensor (APDM, Inc., Portland, OR, USA) on the lumbar spine, as shown in Figure 1. The Opal sensor includes triaxial accelerometers, gyroscopes and magnetometers and records signal data at 128 Hz. To validate the turn detection algorithm, we used Motion Analysis (MA, Santa Rosa, CA, USA) with a set of eight infrared cameras to track reflective markers attached to the pelvis, as well as to the feet. Subjects also wore a sport mini-camera (GoPro, CA, USA) around their waist, pointing at their feet. Subjects were instructed to walk on a path of a mixed route with short straight paths interspersed with ten turns of 45, 90, 135 and 180 degrees in both directions, at three different speeds. Each subject walked the path twelve times: four at a slow speed, four at a preferred speed and four at a fast speed. Inertial data collected in the laboratory was used to develop and validate the turn detection algorithm described in the following section.

### 2.2. Algorithm

Angular rotational rate of the pelvis, measured by the gyroscope about the vertical axis, is an ideal signal to detect turns. The direction of gravity, measured by the accelerometer during a stationary period, can be used to project the gyroscope measurements on to the vertical axis throughout the trial, as described in [33]. In our algorithm, summarized in Algorithm 1, we take advantage of the orientation estimates to obtain angular velocity about the vertical axis using the transformation operation described in Equation (1). Orientation angles are commonly estimated using sensor fusion, taking advantage of the accelerometer measurement of gravity to correct drift from integration of angular velocity measurements [34]. Opal sensors provide orientation estimates *q* in quaternion form and can be used directly to transform body frame sensor measurements into the inertial frame. We transform the angular velocity measurements from the body frame, *ω^b^*, to the inertial frame, *ω^i^*, using the following quaternion multiplication:

(1) $$\omega^{i}=q\times \omega^{b}\times q^{-1}$$

we use this transformed rotational rate *ω^i^* to extract the z component, $\omega_{z}^{i}$, about the vertical axis. This $\omega_{z}^{i}$ is low-pass filtered with a 1.5 Hz cutoff frequency Butterworth filter to remove the high frequency components. Candidate turns are then detected from segments where the maxima of the filtered $\omega_{z}^{i}$ exceed a threshold of 15°/ s. The start and end of each turn are set to the point where the filtered $\omega_{z}^{i}$ drops below 5°/ s. The precise cutoff value has little effect on the total turn duration or angle and is intended to account for the inherent bias in the angular rotational rate.

It is difficult for humans to make more than a very slight turn with a duration <0.5 s or to complete an extremely slow turn with a duration >10 s during gait. Therefore, only turns with durations between 0.5 and 10 s and turn angles over 45° were considered. We combine any turns in the same direction separated by a brief pause <50 ms. Relative turn angles are obtained by integrating $\omega_{z}^{i}$:

(2) $$\theta_{z}=\int \omega_{z}^{i}$$

**Algorithm 1** Turn detection algorithm.
 1:Rotate angular velocity to Earth frame, *ω^i^* 2:Extract vertical component, 
$\omega_{z}^{i}$ 3:Low pass filter 
$\omega_{z}^{i}$ with *f_c_* = 1.5 Hz 4:Find maxima of absolute value, 
$|\omega_{z}^{i}|\geq 15{}^{\circ}/\text{s}$ 5:**for** each maximum **do** 6: find 5°/ s crossings preceding and following maximum 7: turn duration = final zero crossing - initial zero crossing 8:**end for** 9:**for** each turn **do** 10: **if** (intra-turn duration < 0.05 s) and (previous turn is same direction) **then** 11:  combine with previous turn 12: **end if** 13:**end for** 14:**for** each remaining turn **do** 15: **if** (turn duration > 10 s) or (turn duration < 0.5 s) **then** 16:  eliminate turn 17: **end if** 18: turn angle *θ_z_* = Σ*ω_z_* × *T_s_* 19: **if** turn angle *θ_z_* < 45° **then** 20:  eliminate turn 21: **end if** 22:**end for**


### 2.3. Measurement in the Home

**Figure 2.**
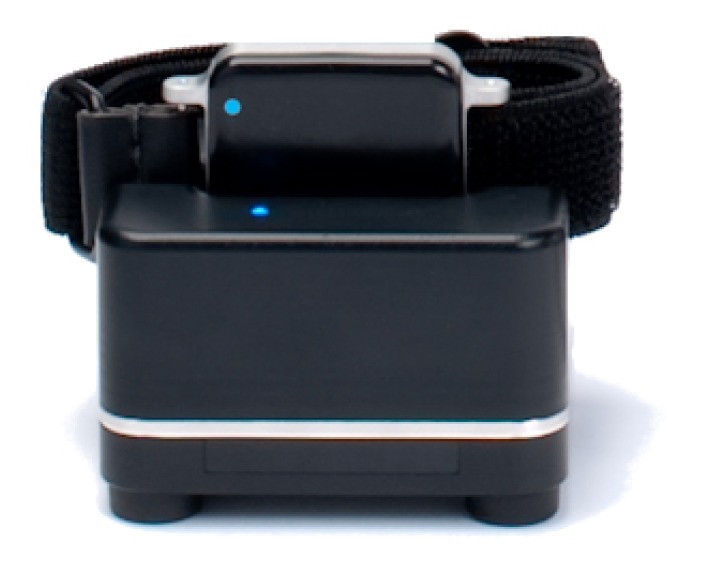
An Opal with a Velcro strap in the docking station to recharge the device.

We enrolled 12 subjects with PD (65 ± 6 years, UPDRS III 24.5 ± 7.5) and 18 older control subjects (86 ± 7.5 years) in the home for seven days. On the morning of the first day, a study coordinator met to wear them and charge them at the end of each day. Three Opal sensors were worn: one on the pelvis at the lumbar vertebral level and one on each foot. The Opal's on-board data storage can hold 720 h worth of data. Opal sensors have sufficient battery life to continuously record data over 16 h throughout the day. Participants wore the Opal sensors all day for the rest of the seven days, recharging them each night. Figure 2 shows an Opal sensor in the docking station used to recharge the device. The sensors are wirelessly synchronized with each other and collect data with a precision of better than ±1 ms. This is important for measuring movement of the feet relative to the trunk during gait and turning.

We expanded the algorithm to capture walking and turning events during spontaneous activities using the data collected in the home. The algorithm identifies periods of walking activities during normal daily activities in the community and calculates the hourly frequency of turning, the duration of each turn, the number of steps needed to complete a turn, the peak and average rotational turning rate and jerk, as well as the variability of these measures throughout the day and week.

Periods of walking (bouts) were detected from the total rotational rate, *ω_t_*, of the lumbar sensor:

(3) $$\omega_{t}=\sqrt{\omega_{x}^{2}+\omega_{y}^{2}+\omega_{z}^{2}}$$

walking bouts were detected when *ω_t_* exceeded a threshold of 15 °/ s for 10 s or longer. Consecutive bouts that were less than 10 s apart were merged together and considered one bout.

The number and duration of each step during detected walking bouts and turns were calculated using the rotational rate data collected from the Opal sensors attached to the subject's feet. For each step, the midpoint of the foot swinging motion (midswing) was first detected by the peak of the pitch angular velocity. Initial and terminal contact of the foot with the ground, marked by the zero-crossing of the pitch angular rate around the midswing, were used to detect each step and its duration.

We us the coefficient of variation (CV) to analyze the variability of the turn metrics throughout the day and week among the PD and control subjects. The CV summarizes the amount of variation as the ratio of the standard deviation, *σ*, to the mean, *μ*.

## 3. Results and Discussion

First, we present the results related to the validation of the inertial sensor algorithms based on optical and video data collected in the laboratory. Then, we compare turning and characteristics between the subjects with and without PD obtained from the continuous inertial data collected in the home.

### 3.1. Validation

**Figure 3.**
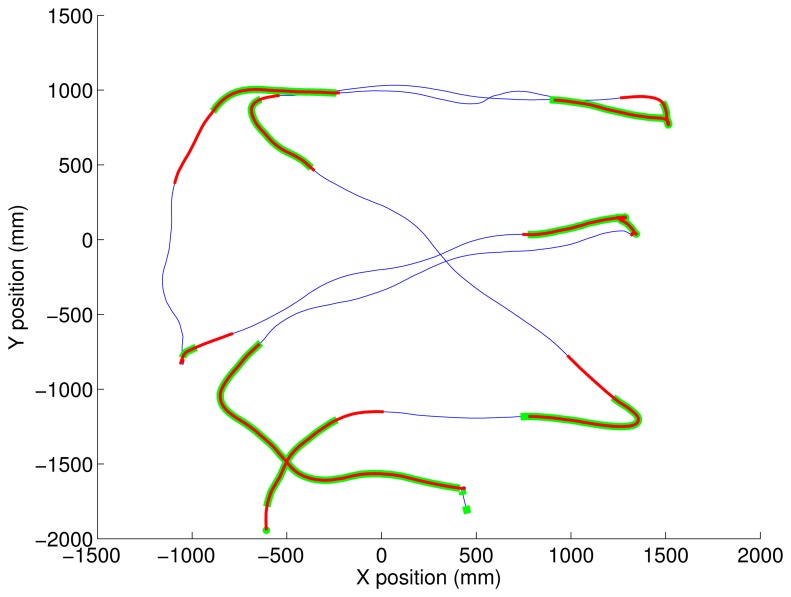
The blue trace is the X-Y position of the body center of mass from the optical markers. Overlaid in red and green are the segments detected as turns by the inertial algorithm and Motion Analysis, respectively.

**Figure 4.**
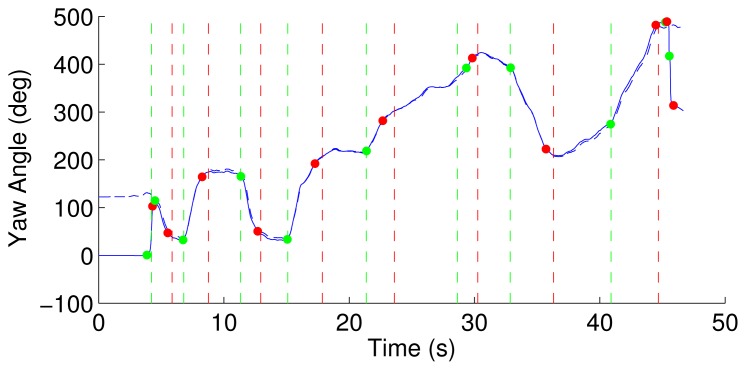
The solid and dashed blue lines represent the yaw angle from Motion Analysis and the inertial algorithm, respectively. The dots and vertical dashed lines represent the onset (green) and end (red) of turns detected by Motion Analysis and the inertial algorithm, respectively.

Figure 3 shows the prescribed path of short straight segments and turns of 45°–180° detected by both Motion Analysis and the inertial algorithm superimposed, as shown in Figure 4.

The video data was reviewed by two independent raters who annotated the beginning and end time of each turn based on the rotation of the feet over the ground. The time indices of turns detected by the two video raters were upsampled from 30 to 128 Hz to match the sampling rate of the inertial data. Similarly, all turn metrics from Motion Analysis were resampled at 128 Hz. Rather than compare the inertial algorithm to video and Motion Analysis based on turn-by-turn detection accuracy, we validate the performance and agreement of the systems based on sample-by-sample accuracy. Although this might degrade the performance results, the timing accuracy of turn detection is important for characterizing turn duration. Therefore, a true positive turn sample was declared when detected by the inertial algorithm and the other gold-standard system. A false negative was declared when a turn sample was detected by the gold-standard system, but not detached by the inertial algorithm.

Table 1 shows the sensitivity of the inertial algorithm detecting turns compared to Motion Analysis and the two video raters. The sensitivity of the inertial algorithm is 0.90 compared with the optical marker algorithm. When compared to the two video raters, the turn detection of the inertial algorithm outperformed the Motion Analysis system. The sensitivity of the inertial algorithm is 0.75 and 0.77 compared to the two video raters, while Motion Analysis has a lower sensitivity of 0.57 and 0.64. The sensitivity of detection between the two video raters was the highest at 0.91.

A true negative was declared when no turn sample was detected by either the inertial algorithm or the gold-standard. A false positive was declared when a turn sample was detached by the inertial algorithm, but not by the gold-standard. The specificity of the inertial algorithm is 0.75, 0.69 and 0.60 with Motion Analysis and the two video raters.

Motion Analysis has a better specificity than the inertial algorithm compared to the video raters; see Table 2.

Despite the good agreement between the inertial algorithm and the other gold-standard systems, some disagreement could be attributed to the different definition of a turn used with each system. For example, the video raters were instructed to annotate the time of the heel strike prior to and following the end of each turn. The heel strikes were chosen because the onset and end of each turn starting at the subject's waist is often ambiguous from the video recording. This, however, differs from the way that humans tend to turn by first rotating the pelvis around the vertical axis before turning their feet. This is consistent with the turn definition used for the inertial algorithm, which is based on the angular velocity of the lumbar. The optical marker-based algorithm is qualitatively similar, in that it first estimates a continuously varying yaw angle and then detects turns based on the turn angle. This might explain the better agreement between the Motion Analysis and inertial detected turns.

The inertial sensor-based algorithm can be used to detect turns during walking at least as well as the Motion Analysis system. The number of detected turns for the inertial algorithm averaged 10.9 ± 1.6 across all subjects and trials. Similarly, the algorithm based on optical markers detected an average of 10.4 ± 3.4 turns. Subjects were inconsistent with one particular turn, when they were asked to pick up a light basket and turn. Some subjects avoided the turn altogether by picking up the basket without any turning at the waist. In this close pattern with the rapid series of turns, some subjects blended two turns together as they transitioned fluidly from one to the next rather than following the line precisely. Both of these factors contribute to variability in the number of detected turns, especially in the fast trials.

Tables 3 and 4 show turn metrics detected by the inertial algorithm, including the average number of turns, turn peak and mean velocity in degrees per seconds and turn duration in seconds for control and PD subjects.

Analysis of the pelvic rotation shows that PD subjects had a slower velocity compared to controls while turning at their self-determined slow, preferred and faster gait speeds. Even when matched for the same gait speed, patients with PD show impaired turning because of the significant challenge to their dynamic balance. However, turn duration was not significantly different between PD and control subjects.

It has been shown that PD patients appear to walk better when they are examined in an outpatient clinic or in a research laboratory than caregivers report about their daily lives [35]. This is known as the “white coat” effect. Hence, turn detection and characterization in the home and community is important for continuous assessment of gait and balance in people with movement disability.

### 3.2. Turn Metrics in the Home

**Figure 5.**
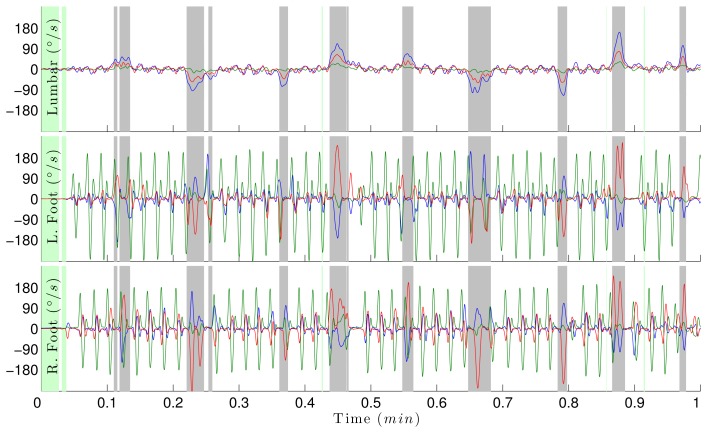
Rotational rate of the inertial sensors (Opals) attached to the lumbar (**top**), left foot (**middle**) and right foot (**bottom**). Blue, green and red traces are the x-, y- and z-axes of the gyroscope in degrees per second. Green areas represent periods in which the subject is not walking; gray represents periods of turning, and white areas represent periods of walking.

Using the inertial algorithm validated with the laboratory data, we analyze the continuous data collected in the home. We expanded the algorithm to capture walking and turning events during spontaneous activities. The algorithm identifies periods of walking activities inside and outside the home and calculates the number of turns and corresponding turn metrics during each hour of the day. Figure 5 shows how gyroscope data can be used to automatically identify periods of turning, walking and standing over a minute of continuous monitoring. The figure shows the rotational rate of the sensors attached to the lumbar, left foot and right foot. A turn is detected when the total rotational rate of the lumbar sensors exceeds the threshold, shown by the gray areas representing periods when the subject is turning.

Table 5 shows the average number of bouts per hours (No. Bouts/h) for control and PD subjects, bout and step duration in seconds and their coefficient of variation (CV), as well as active rate. The table also shows the *p*-value for a *t*-test at a 5% level of significance to determine if PD and CT bout and turn metrics significantly differ from each other. Active rate is defined as the percentage of time when the subject is walking or turning compared to the full time of monitoring during the day. Bout duration and active rate are statistically significantly different between PD and control subjects. Active rate and number of bouts per hour show the largest variation among all subjects, with a clear trend of subjects being more active during the first three hours of the day. Activity rate declines for an average of three hours, then increases again during the 7–8th h. PD and control subjects generally complied with wearing the inertial sensors as instructed for the entire week, for an average of ten hours per day.

Table 6 shows the average number of turns per hour, turn duration in seconds, average turn angle and peak velocity and the number of steps to complete a turn, as well as their coefficient of variation. The table also shows the *p*-value for the *t*-test.

Subjects exhibit a great variability of the number of turns throughout the day, with an average of 65.2 and 67.3 turns/h for control and PD subjects, respectively. PD subjects tend to complete shorter turns with smaller turn angles, compared to the control group. Data also shows that PD subjects complete a turn with a larger number of steps. Consistent with the results obtained from the laboratory data, PD subjects show more variability than controls in all turn metrics throughout the day and the week.

Compared to turn duration during the prescribed tasks in the laboratory (1.4 s), both control and PD subjects completed a turn with a longer average duration of 2.2 s and 2.0 s, respectively. Similarly, analysis of the pelvic rotational rate during turning shows that subjects significantly decreased their rotational rate in the home, compared to in the laboratory. In a previous study, it was demonstrated that even an identical instrumented test of mobility conducted in the home environment resulted in slower turns and walking than when conducted in the laboratory [36].

## 4. Conclusions

In this study, we developed a wearable inertial system for continuous monitoring to detect and distinguish turning characteristics in healthy subjects and individuals with Parkinson's disease (PD). Assessment of the system in the laboratory showed that the inertial system can be used to detect turns with a comparable accuracy to that of the gold-standard Motion Analysis. We demonstrated that we could use the inertial system to measure locomotor activities and to characterize turns in the home throughout the day and week, using three wearable sensors. The algorithm successfully quantifies turns, and results show significant differences between turn characteristics of PD and control subjects. Compared to controls, PD subjects tend to take shorter turns with smaller turn angles and more steps. Furthermore, PD subjects show more variability in all turn metrics throughout the day and the week. Precise measures of turning mobility will improve intervention for balance disorders and fall prevention in the elderly and in patients with neurological diseases.

## Figures and Tables

**Table 1.** Sensitivity.

	**Motion Analysis**	**Video-Rater 1**	**Video-Rater 2**
Inertial	0.90	0.75	0.77
Motion Analysis		0.57	0.64
Video-Rater 1			0.91

**Table 2.** Specificity.

	**Motion Analysis**	**Video-Rater 1**	**Video-Rater 2**
Inertial	0.75	0.69	0.60
Motion Analysis		0.77	0.73
Video-Rater 1			0.72

**Table 3.** Control turn metrics. CV, coefficient of variation.

**Metric**	**Slow (CV)**	**Normal (CV)**	**Fast (CV)**
# of turns	11.7 (0.16)	12.0 (0.10)	8.9 (0.20)
Peak Velocity (deg/s)	117.1 (0.12)	131.9 (0.12)	165.9 (0.15)
Mean Velocity (deg/s)	69.4 (0.13)	78.0 (0.15)	82.5 (0.16)
Duration (s)	1.4 (0.07)	1.4 (0.14)	1.7 (0.18)

**Table 4.** PD turn metrics.

**Metric**	**Slow (CV)**	**Normal (CV)**	**Fast (CV)**
# of turns	11.8 (0.14)	11.8 (0.14)	9.7 (0.15)
Peak Velocity (deg/s)	109.6 (0.20)	124.8 (0.29)	146.7 (0.18)
Mean Velocity (deg/s)	66.6 (0.15)	69.7 (0.18)	75.4 (0.17)
Duration (s)	1.4 (0.14)	1.3 (0.15)	1.5 (0.20)

**Table 5.** Bout metrics. CT, control.

	**No. Bouts/h (CV)**	**Duration (CV)**	**Step Duration (CV)**	**Active-Rate (CV)**
CT	12.1 (0.73)	61.1 (0.47)	1.1 (0.38)	20.2 (0.88)
PD	12.3 (0.71)	70.6 (0.45)	1.1 (0.40)	23.7 (0.90)
*p-value*	0.610	0.001	0.057	0.001

**Table 6.** Turn metrics.

	**No. Turns/h (CV)**	**Duration (CV)**	**Angle (CV)**	**Peak Velocity (CV)**	**No. Steps (CV)**
CT	65.2 (0.89)	2.2 (0.23)	95.2 (0.15)	73.2 (0.26)	3.1 (0.23)
PD	67.3 (.92)	2.0 (0.38)	92.0 (0.19)	76.7 (0.28)	3.5 (0.24)
*p-value*	0.445	0.001	0.001	0.002	0.001
